# Modeling Propulsion of Soft Magnetic Nanowires

**DOI:** 10.3389/frobt.2020.595777

**Published:** 2020-10-29

**Authors:** Yoni Mirzae, Boris Y. Rubinstein, Konstantin I. Morozov, Alexander M. Leshansky

**Affiliations:** ^1^Department of Mathematics, Technion–Israel Institute of Technology, Haifa, Israel; ^2^Stowers Institute for Medical Research, Kansas City, MO, United States; ^3^Department of Chemical Engineering, Technion–Israel Institute of Technology, Haifa, Israel; ^4^Russel Berrie Nanotechnology Institute, Technion–Israel Institute of Technology, Haifa, Israel

**Keywords:** microswimmer, micropropeller, magnetic nanowire, driven propulsion, flexible filament, bead-spring model

## Abstract

The emergent interest in artificial nanostructures that can be remotely navigated a specific location in a fluidic environment is motivated by the enormous potential this technology offers to biomedical applications. Originally, bio-inspired micro-/nanohelices driven by a rotating magnetic field were proposed. However, fabrication of 3D helical nanostructures is complicated. One idea to circumvent complex microfabrication is to use 1D soft magnetic nanowires that acquire chiral shape when actuated by a rotating field. The paper describes the comprehensive numerical approach for modeling propulsion of externally actuated soft magnetic nanowires. The proposed bead-spring model allows for arbitrary filament geometry and flexibility and takes rigorous account of intra-filament hydrodynamic interactions. The comparison of the numerical predictions with the previous experimental results on propulsion of composite two-segment (Ni-Ag) nanowires shows an excellent agreement. Using our model we could substantiate and rationalize important and previously unexplained details, such as bidirectional propulsion of three-segment (Ni-Ag-Au) nanowires.

## 1. Introduction

Development of artificial nanomachines that can controllably propel through complex fluidic environments is one of the most exciting challenges of nanotechnology. The emergent approaches range from catalytically driven nanowires, Janus particles to thermally, light- and ultrasound-driven colloids (Wang, 2013). One of the promising methods is magnetic actuation. While the traditional techniques are based on strong gradient magnetic fields to generate a *force* for remote towing of magnetic nanoparticle, the alternative approach relies on a weak (milli Tesla) uniform rotating magnetic field that serves to apply a *torque* twirling the nanomotor. Given that the particle shape admits non-trivial rotation-translation coupling, such torque-driven twirling will result in net propulsion. Notice that while the field gradient can tow an isotropic (i.e., spherical) particle, it is not very efficient due to the need of large field strength (of the order of Tesla) required for creating appreciable variance of the field at the size of the nanoparticle. Although propulsion based on external magnetic torque requires more complex particle shape [e.g., helical (Ghosh and Fischer, 2009; Zhang et al., 2009)], it offers a remote, fuel-free and engineless propulsion in a variety of fluidic environments with typical velocities considerably exceeding the speed of gradient towing.

This technology has been extensively studied over the last decade by a number of groups. Various methods, such as “top-down” approach (Zhang et al., 2009), delamination of magnetic stripes (Smith et al., 2011), glancing angle deposition Ghosh and Fischer (2009), direct laser writing (Tottori et al., 2012), biotemplated synthesis using biological spiral organelles (Gao et al., 2014), two-photon polymerization of a curable magnetic polymer composite (Peters et al., 2013), spiraling microfluidic flow lithography (Yu et al., 2017), and other techniques have been developed for fabrication of μm-size and sub-μm-size (Schamel et al., 2014) helical motors. These bio-inspired helical motors propel *unidirectionally* (along the field rotation axis) when driven by a rotating magnetic field similar to a twirling bacterial flagellum. However, fabrication of three-dimensional (3D) chiral/helical nanostructures requires sophisticated procedures and the search for simpler alternatives is under way.

One alternative relies on the fact that in torque-driven propulsion the propeller's shape may not necessarily be helical or even chiral. It was recently demonstrated that geometrically *achiral* objects made of three interconnected magnetized microbeads can be steered quite efficiently by an in-plane rotating magnetic field (Cheang et al., 2014). These findings suggested that the two-dimensional (2D) ferromagnetic propellers could be of practical interest, as they can be mass-fabricated via standard photolithography methods (Tottori and Nelson, 2018). It was theoretically predicted (Morozov et al., 2017) and demonstrated experimentally [using up-scaled cm-size propeller (Sachs et al., 2018)] that unidirectional propulsion of 2D magnetic structures is feasible, however it requires non-trivial off-plane magnetization. Since 2D structures are prone to magnetize in-plane, uniform off-plane magnetization at nano/microscale cannot be easily achieved.

Another possibility to avoid sophisticated microfabrication relies on spontaneous aggregation of magnetic nanoparticles into random-shaped 3D microclusters (Vach et al., 2013, 2015). These random aggregates can also be steered through fluid by an external torque, however they appear to be significantly less efficient “swimmers” on average in comparison to nanomotors with preprogrammed (optimal) geometry and magnetization (Mirzae et al., 2018).

There is an additional possibility that uses even simpler one-dimensional (1D) *soft* magnetic nanowires. The first demonstration of soft artificial “swimmer” was provided by Dreyfus et al. (2005) whereas a linear chain of magnetic microbeads linked by DNA and attached to a red blood cell was actuated by a plane oscillatory magnetic field. This “swimmer” was undergoing in-plane undulations and propelled similar to a flagellum of an eukaryotic cell. A minimal design of the planar undulating magnetic microswimmer made of just two rigid links connected by a torsional spring was suggested by Gutman and Or (2015) and the corresponding nanowire-based analog was demonstrated by Jang et al. (2015). Highly efficient two-arm magnetic nanoswimmer exhibiting complex 3D (“freestyle”) undulations driven by an in-plane oscillatory magnetic field was reported in Li et al. (2017).

Flexible 1D nanowire-based propellers steered by a rotating field were reported by Gao et al. (2010). These nanomachines were fabricated by electrodeposition from a composite nanowire that had a rigid magnetic (Ni) head, flexible (porous Ag) middle segment and passive rigid (Au) tail. The flexible segment deforms and supposedly acquires helicity due to an interplay of viscous and elastic forces when actuated by rotating magnetic field and the nanowire propels similar to the 3D rigid helical motors. The nanowire propeller in (Gao et al., 2010) had a total length 6.5 μm and diameter 100 nm and exhibited propulsion with speed of ~5 μm/s when actuated by the magnetic field rotating in-plane with frequency 10–15 Hz. Pak et al. (2011) proposed a similar two-segment (Ni-Ag) design whereas nanowire propeller was driven by a *conically* rotating magnetic field (permanent magnetic field applied along the axis of the rotating field). This Ni-Ag propeller showed even faster propulsion in comparison to Gao et al. (2010). Interestingly, the three-segment nanowire of Gao et al. (2010) could propel either head-forward (fast) or tail-forward (slow), whereas the two-segment nanowire in Pak et al. (2011) could only propel head-forward.

Theoretical modeling of soft nanowire propellers is quite limited. Gauger and Stark (2006) put forward a discrete bead-spring model for simulating externally driven undulatory propulsion of a flexible filament powered by an oscillating magnetic field reported by Dreyfus et al. (2005). Only planar undulations (via bending) were considered and the hydrodynamics was modeled using approximate Rotne-Prager method. Pak et al. (2011) put forward an approximate elastohydrodynamic model based on the dynamic deformation of initially straight elastic filament that is steadily rotated at its one (clamped) end, while the other end is free. The filament is rotated in a way, that its long axis forms a constant conical angle with the rotation axis mimicking actuation by a conically rotating magnetic field. Although this model showed a reasonable agreement with the experiments, it only imitates the real problem in which both ends of the nanowire are free. Various analytical theories concerning two-, three-, or multi-link propellers take advantage of simplified (local) hydrodynamics that neglects hydrodynamic interaction between the rigid links (see e.g., Gutman and Or, 2015; Jang et al., 2015; Alouges et al., 2019).

In the present paper we develop a numerical approach for modeling of flexible, semi-flexible or multi-link externally driven nanopropellers. The numerical algorithm is based on a discrete bead-spring model of the filament. Multipole Expansion (ME) algorithm allows for rigorous account of non-local intra-filament hydrodynamic interaction mediated by a viscous fluid, while springs connecting the beads model the elastic deformation (due to bending and torsion). The ability to accurately model viscous hydrodynamic forces is important for accurate simulations of non-slender objects and large-amplitude deformations (see e.g., Berman et al., 2013 for the importance of non-local hydrodynamics in locomotion powered by large-amplitude undulations). This is particularly relevant for externally actuated nanowire propellers, whereas the deformation is not known in advance and determined by an interplay of elastic, magnetic and hydrodynamic forces. Below we demonstrate the applicability of the proposed approach toward simulations of soft nanowire-based propellers (Gao et al., 2010; Pak et al., 2011).

## 2. Results

### 2.1. Bead-Spring Model of the Flexible Filament

#### 2.1.1. Elastic and Magnetic Energies

**Figure 1:**
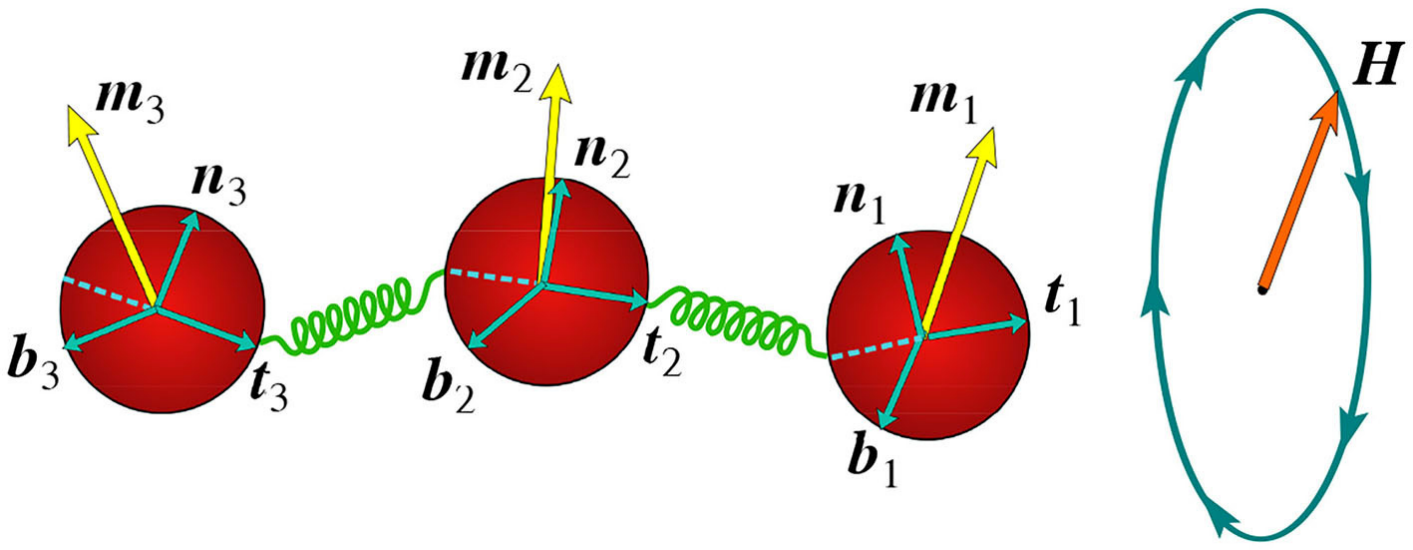
Illustration of the bead-spring model: magnetized beads connected by springs actuated by an in-plane rotating magnetic field. Triads of local orthogonal unit vectors {*b*_*i*_, *n*_*i*_, *t*_*i*_} affixed to each bead together with magnetic moments *m*_*i*_ are showing.

The bead-spring model of the flexible filament comprises of *N* identical spherical beads of radius *a* (beads of different radii *a*_*i*_ can also be used) connected by *N* − 1 springs. Each bead is characterized by its position *r*_*i*_ and a triad of orthogonal (binormal, normal, and tangent) unit vectors {*b*_*i*_, *n*_*i*_, *t*_*i*_} (Maggs, 2000; Wada and Netz, 2012), see the schematic illustration in Figure 1. The *i*th bead is linked to its neighbors by springs connected to its surface at points *r*_*i*_ − *a*_*i*_*t*_*i*_ and *r*_*i*_ + *a*_*i*_*t*_*i*_.

There are three contributions into the elastic energy: stretching, bending and torsion, $E^{el}=E_{st}+E_{b}+E_{t}$. The stretching energy reads

(1) $$E_{st}=\frac{K}{2}\overset{N-1}{\underset{i=1}{\sum }}{\left(|r_{i}-a_{i}t_{i}-r_{i+1}-a_{i+1}t_{i+1}|-l_{0}\right)}^{2},$$

where *K* is the spring constant and *l*_0_ is the length of the spring at rest. The constant *K* is assumed to be sufficiently large to preserve the curvilinear length of inextensible or weakly extensible filament. We use non-zero values of the rest lengths *l*_0_ to prevent the beads from overlapping and avoid the necessity to introduce steric repulsions between neighboring beads. In calculations we typically used the values *l*_0_ ~ 0.25*a*. Notice that *K* should not necessarily be constant along the filament. When the filament is made of several segments (e.g., of different rigidity), the value of the spring constant can be prescribed separately to each segment.

The bending energy describes the preferable orientation of the tangential vector *t*_*i*_ with respect to the line of centers *r*_*i,i*+1_ connecting the neighboring beads. The characteristic bending energy of a bead-spring contact is $\frac{A}{a}\left(1-t_{i}\cdot {\hat{r}}_{i,i+1}\right)$, where *A* is the bending modulus and ${\hat{r}}_{i,i+1}$ is the unit vector along the line of centers of *i* and *i* + 1 beads, ${\hat{r}}_{i,i+1}=\frac{r_{i}-r_{i+1}}{\left|r_{i}-r_{i+1}\right|}$. Summing up all bead-spring contacts and symmetrizing the bending energy relative to both filament ends, one obtains

(2) $$E_{b}=\frac{A}{2a}\overset{N-1}{\underset{i=1}{\sum }}\left[2-\left(t_{i}+t_{i+1}\right)\cdot {\hat{r}}_{i,i+1}\right],$$

where *A* is the bending modulus.

Notice that the bending modulus *A* was chosen in such a way that the relation (2) would reduce to the form $E_{b}=\left(A/2a\right)\underset{i}{\sum }\left(1-t_{i}\cdot t_{i+1}\right)$ commonly used for chains of beads in contact (i.e., for *l*_0_ = 0) (Maggs, 2000; Wada and Netz, 2012), for small deviations of *t*_*i*_ and *t*_*i*+1_ from the vector *r*_*i, i*+1_. Let us choose the local spherical coordinate system with the polar axis aligned with the unit vector ${\hat{r}}_{i,i+1}$, i.e., ${\hat{r}}_{i,i+1}=\left(0,0,1\right)$. In this frame the vectors *t*_*i*_ and *t*_*i*+1_ take the form *t*_*i*_ = (sinθ_*i*_cosϕ_*i*_, sinθ_*i*_sinϕ_*i*_, cosθ_*i*_) and *t*_*i*+1_ = (sinθ_*i*+1_cosϕ_*i*+1_, sinθ_*i*+1_sinϕ_*i*+1_, cosθ_*i*+1_). Neglecting the cross terms in the scalar product *t*_*i*_·*t*_*i*+1_, we have $t_{i}\cdot t_{i+1}≅\cos\theta_{i}\cos\theta_{i+1}\approx 1-\frac{1}{2}\theta_{i}^{2}-\frac{1}{2}\theta_{i+1}^{2}$. The expression in the square brackets in Equation (2) reads $2-\left(t_{i}+t_{i+1}\right)\cdot {\hat{r}}_{i,i+1}=1-\cos\theta_{i}-\cos\theta_{i+1}$$\approx \frac{1}{2}\theta_{i}^{2}+\frac{1}{2}\theta_{i+1}^{2}$. Thus, in the limit of small deformations we have $2-\left(t_{i}+t_{i+1}\right)\cdot {\hat{r}}_{i,i+1}\approx {1-t}_{i}\cdot t_{i+1}$.

The torsional energy can be written in the form (Maggs, 2000; Wada and Netz, 2012)

(3) $$E_{t}=\frac{C}{4a}\overset{N-1}{\underset{i=1}{\sum }}\left(1-n_{i}\cdot n_{i+1}-b_{i}\cdot b_{i+1}+t_{i}\cdot t_{i+1}\right),$$

where *C* is the twisting modulus. The coefficients *A* and *C* have the units of energy times length, *K* has units of energy per area, while their respective magnitudes typically satisfy *Ka*^3^ ≫ *A* ≈ *C*.

The magnetic energy is owing to the interaction of the net magnetic moment $m=\underset{i}{\sum }m_{i}$ (see in Figure 1) with the external magnetic field *H*

(4) $$E_{m}=-m\cdot H.$$

Magnetic moment of an individual bead, *m*_*i*_, is affixed to the bead coordinate system formed by the triad {*n*_*i*_, *b*_*i*_, *t*_*i*_}. Assuming these vectors to be the {*x, y, z*} axes of the local coordinate system, we characterize the local orientation of *m*_*i*_ by two spherical angles θ_*m*_*i*__ and ϕ_*m*_*i*__, *m*_*i*_ = *m*_*i*_{sinθ_*m*_*i*__cosϕ_*m*_*i*__, sinθ_*m*_*i*__sinϕ_*m*_*i*__, cosθ_*m*_*i*__}.

#### 2.1.2. Force and Torque Balances

The elastic force $F_{i}^{el}$ acting on the *i*th bead is derived from Equations (1) and (2) as

(5) $$F_{i}^{el}=-\frac{\partial E_{st}}{\partial r_{i}}-\frac{\partial E_{b}}{\partial r_{i}}.$$

The force $F_{i}^{el}$ has two contributions $F_{i}^{el}=F_{i-1}^{el}+F_{i+1}^{el}$, where $F_{i-1}^{el}$ and $F_{i+1}^{el}$ are the elastic forces exerted on the *i*th bead by its neighbors, i.e., (*i* − 1)th and (*i* + 1)th beads, respectively. The corresponding torque *L*_*i*_ exerted on the *i*th bead is a sum of the magnetic $L_{i}^{m}$ and elastic $L_{i}^{el}$ contributions, $L_{i}=L_{i}^{m}+L_{i}^{el}$, where

(6) $$L_{i}^{m}=m_{i}\times H$$

and (Espanol, 1998; Wada and Netz, 2012)

(7) $$\begin{array}{l} L_{i}^{el}=-n_{i}\times \frac{\partial E^{el}}{\partial n_{i}}-b_{i}\times \frac{\partial E^{el}}{\partial b_{i}}-t_{i}\times \frac{\partial E^{el}}{\partial t_{i}}-\frac{r_{i-1,i}}{2}\times F_{i-1}^{el}\\ \;+\frac{r_{i,i+1}}{2}\times F_{i+1}^{el}. \end{array}$$

The elastic forces and magnetic and elastic torques acting on each bead are balanced by the corresponding hydrodynamic (viscous) forces $F_{i}^{h}$ and torques $L_{i}^{h}$:

(8) $$F_{i}^{el}+F_{i}^{h}=0,\;L_{i}^{el}+L_{i}^{m}+L_{i}^{h}=0.$$

The viscous forces and torques applied to each bead are determined via the multipole expansion (ME) method (Filippov, 2000) that allows careful account of the mutual hydrodynamic interactions between different beads composing the filament. The method relies on multipole expansion of the Lamb's spherical harmonic solution of the Stokes equations in the unbounded fluid (Filippov, 2000). Given *N* spheres with radii *a*_*i*_, the algorithm outputs the translational and angular velocities of the spheres for the input of prescribed forces and torques acting on each sphere. The no-slip condition at the surface of all spheres is enforced rigorously via the use of direct transformation between solid spherical harmonics centered at origins of different spheres. The ME method solves a system of $O\left(NL^{2}\right)$ linear equations for the expansion coefficients and velocities, whereas the accuracy is controlled by the truncation level, L, equal to the number of spherical harmonics retained in the expansion. The validity and accuracy of the ME algorithm have been previously tested for (i) the exact solution (in bi-spherical coordinates) for the flow past two close spheres and against (ii) a boundary element method numerical solution for the translation and rotation of straight chains of spheres (made of *N* = 2–30 spheres, Filippov, 2000). The method was previously applied for modeling self-locomotion of undulating filament (Berman et al., 2013), and externally driven propulsion of a microhelix (Walker et al., 2015), arc-shaped filament (Morozov et al., 2017; Sachs et al., 2018), and random fractal-like aggregates (Mirzae et al., 2018). In our calculations the truncation level was set to L=2–3, as it yielded sufficiently accurate results, while keeping the small size of the linear system to be solved at each time step. Notice that the extension of the ME method to wall-bounded domain is possible (Ozarkar and Sangani, 2008). The applied ME method valid for unbounded fluid domain can also be used for modeling propulsion near boundary by adding a stationary sphere of a large radius (e.g., larger than the filament length) in the vicinity of the micro-swimmer.

#### 2.1.3. Non-dimensionalization

In the numerical simulations we distinguish between the parameters for the rigid and flexible segments of the filament. In particular, the elasticity constants of the rigid segments are much higher than the corresponding parameters for the flexible part. Typically the magnetic Ni segment is rigid, and thus its net magnetic moment *m* can be distributed equally between beads comprising it.

For simplicity we assume uniform thickness of the filament, i.e., the equal-sized beads with radius *a* composing the rigid and flexible parts. For characteristic scales of length, time and elasticity we use, respectively, the bead radius *a*, the period of the rotating field *T* = 2π/ω and the twisting modulus *C* of the flexible segment.

The non-dimensional form of the forces and torques balances in Equation (8) read,

(9) $${\hat{F}}_{i}^{h}=-\frac{1}{p}\left(C_{st}{\hat{F}}_{i}^{st}+C_{b}{\hat{F}}_{i}^{b}\right),$$

(10) $${\hat{L}}_{i}^{h}=-\frac{1}{p}\left(C_{st}{\hat{L}}_{i}^{st}+C_{b}{\hat{L}}_{i}^{b}+{\hat{L}}_{i}^{t}+C_{m}{\hat{L}}_{i}^{m}\right),$$

where the corresponding ${\hat{F}}_{i}$ and ${\hat{L}}_{i}$ stand for the dimensionless hydrodynamic (superscript *h*), bending (*b*), stretching (*st*), and magnetic (*m*) forces and torques, exerted on *i*th bead. Here *p* = ω/ω_*f*_ is a ratio of the frequency of the magnetic field, ω, and the characteristic frequency of the filament, $\omega_{f}=\pi C/\eta a^{4}$, where η is the dynamic viscosity of the fluid. *C*_*m*_ = 2*amH*/*C* measures the relative magnitude of the elastic and magnetic moments, $C_{st}=2Ka^{3}/C$ stands for the ratio of stretching and twisting coefficients and *C*_*b*_ = *A*/*C* for the ratio of bending and twisting coefficients. Typically we assume that *C*_*b*_ ≈ 1 (Landau and Lifshitz, 1970) and *C*_*st*_ ≫ 1. The last condition implies nearly incompressible filament. Notice that the twist modulus *C* of the *flexible* part of the filament was chosen for non-dimensionalization. The observable deformation of the flexible filament in the external magnetic field takes place when the magnitudes of characteristic magnetic and elastic torques are close, i.e., *C*_*m*_ ~ 1. For the *rigid* segment(s) the corresponding value of *C*_*m*_ is smaller by a factor *C*/*C*_*rigid*_ ≪ 1.

The forces and torques exerted on a bead in Equations (9) and (10) are proportional to the rigidity of its links and to *p*^−1^. Therefore, in cases where the composite nanowire has rigid segment(s) (with *p* ≪ 1) composed of multiple beads, the equations of time-evolution become stiff, forcing a small time step Δ*t* and prolonged computation time. To overcome this difficulty, we assume that the rigid (small-*p*) section does not deform and participate in a rigid-body motion. The unknown velocities of the individual beads comprising the rigid section are expressed in terms of the translation and rotation velocity of the segment as a whole. In addition, the net hydrodynamic force and torque acting on the rigid segment are calculated as

(11) $${\hat{F}}_{rigid}=\underset{i}{\sum }{\hat{F}}_{i}^{h},$$

(12) $${\hat{L}}_{rigid}=\underset{i}{\sum }\left({\hat{L}}_{i}^{h}+R_{i}\times {\hat{F}}_{i}^{h}\right),$$

where *i* iterates over the beads composing the rigid part and *R*_*i*_ is the vector connecting a central point of the segment and the center of the *i*th bead. This modification removes the need to compute negligibly small deformations due to stiff elastic links. Furthermore, for every rigid segment made of *M* beads, the number of unknown variables in the linear system is reduced by 6(*M* − 1). Using this method we found acceleration of up to ~20 times in simulation speed depending on the value of *p* in comparison with the scheme that treats both rigid and flexible segments similarly based on Equations (9) and (10).

#### 2.1.4. Initial Setup and Time-Evolution

Consider the laboratory coordinate system with the axes {*X, Y, Z*}. Initially the filament is at rest, parallel to the *Z*-axis, so as at *t* = 0, all tangent unit vectors are *t*_*i*_ = (0, 0, 1). We assume the initial orientation of the binormal *b*_*i*_ and normal *n*_*i*_ vectors along the axes *X* and *Y*, respectively: *b*_*i*_ = (1, 0, 0) and *n*_*i*_ = (0, 1, 0). As was mentioned above, the orientation of the magnetic moment of *i*th magnetized bead, ${\hat{m}}_{i}=m_{i}/m$, is prescribed by the two spherical angles θ_*m*_*i*__ and ϕ_*m*_*i*__ and initial orientation of the net magnetic moment is given by a superposition $\underset{i}{\sum }{\hat{m}}_{i}$.

The time-varying (e.g., rotating or oscillating) external magnetic field *H* is switched on at the moment *t* = 0. Since the initial geometry is prescribed, one can determine the forces *F*_*i*_ from (5) and torques *L*_*i*_ from (6–7) exerted on all beads. Then equating the respective elastic and hydrodynamic forces and torques one finds the translational *U*_*i*_ and angular Ω_*i*_ velocities of beads at first time step by using the ME algorithm.

Using these values we obtain the new positions and orientations of beads at the moment *t* = Δ*t*:

(13) $$r_{i}\left(\Delta t\right)=r_{i}\left(0\right)+U_{i}\Delta t,$$

(14) $$\xi_{i}\left(\Delta t\right)=\xi_{i}\left(0\right)+\left[\Omega_{i}\times \xi_{i}\left(0\right)\right]\Delta t,$$

whereas ξ_*i*_ stands for either *b*_*i*_, *n*_*i*_ or *t*_*i*_. Since the magnetic moment of all magnetic beads is affixed to the triad {*b*_*i*_, *n*_*i*_, *t*_*i*_}, its time-evolution is also governed by an equation similar to (14) whereas ξ_*i*_ is replaced by ${\hat{m}}_{i}$ everywhere. The updated vectors according to Equation (14) are normalized with |ξ_*i*_| to keep their magnitude of one. At the next time step the procedure is repeated.

### 2.2. Validation

We tested the validity of the proposed numerical model by comparing its results to two benchmark problems for which theoretical predictions exist: (i) magnetized rigid cylinder actuated by a rotating magnetic field; (ii) the twirling-whirling instability of an elastic filament rotated by its end. The first problem tests the hydrodynamic part of the algorithm, as filament is assumed to be rigid, while the second problem concerns the interplay between elasticity and hydrodynamics.

#### 2.2.1. Magnetized Rigid Cylinder in a Rotating Field

Consider the dynamics of a cylinder with magnetic moment *m* driven by a rotating uniform magnetic field *H* = *H*(cosω*t*, sinω*t*, 0): at low frequency ω the cylinder tumbles in the plane of the field rotation in-sync with the field; at a certain critical frequency, $\omega_{c}^{\left(I\right)}$ (or the corresponding dimensionless parameter $p_{c}^{\left(I\right)}$), the tumbling switches to in-sync wobbling, where the precession angle (i.e., the angle between the field rotation axis and the long axis of the cylinder) gradually diminishes as the frequency is increased; then, at the step-out frequency $\omega_{c}^{\left(II\right)}$ ($p_{c}^{\left(II\right)}$) the synchronous regime switches to the asynchronous one. The analytical solution of the problem was given in Morozov and Leshansky (2014). The dynamics of a cylinder-like structure of a linear chain composed of 5 beads (see Supplementary Material for details) was simulated, yielding the numerical value of the angle α at steady-state between the vectors *m* and *H*. The ratio of the longitudinal and transverse components of the cylinder's magnetization *m* was set to *m*_|_|/*m*_⊥_ = 2.33, and the dimensionless parameters were set to *C*_*st*_ = 2, *C*_*b*_ = 1, and *C*_*m*_ = 0.0015, resulting in the critical values $p_{c}^{\left(I\right)}\approx 2.48\cdot 10^{-7}$ and $p_{c}^{\left(II\right)}\approx 8.70\cdot 10^{-7}$. Table 1 shows a comparison of the theoretical values of α in the tumbling and wobbling regimes given, respectively by Equations (16) and (18) in Morozov and Leshansky (2014), to the numerical values obtained in this work. The agreement between the theoretical predictions and the numerical results is excellent.

**Table 1:** The angle α between the magnetic moment *m* of a linear chain made of 5 beads and the rotating magnetic field *H* in the in-synch tumbling (upper table) and wobbling (lower table) regimes.

**$0<p<p_{c}^{\left(I\right)}(\times 10^{7})$**	**α** **(rad)**	
	**Equation (16) (Morozov and Leshansky**, **2014****)**	**This work**
**In-synch tumbling**		
0.31	0.125	0.125
0.62	0.253	0.253
1.24	0.525	0.525
1.86	0.851	0.851
2.29	1.178	1.165
**$p_{c}^{\left(I\right)}<p<p_{c}^{\left(II\right)}(\times 10^{7})$**	**α** **(rad)**	
	**Equation (18) (Morozov and Leshansky**, **2014****)**	**This work**
**In-synch wobbling**		
2.49	1.168	1.168
3.11	1.179	1.178
3.73	1.192	1.191
4.35	1.208	1.206
4.97	1.227	1.225
5.59	1.250	1.248
6.22	1.277	1.273
6.84	1.310	1.305
7.46	1.352	1.345

*The critical p values at the tumbling-to-wobbling transition and the step-out (synchronous-to-asynchronous transition) are $p_{c}^{\left(I\right)}\approx 2.48\cdot 10^{-7}$ and $p_{c}^{\left(II\right)}\approx 8.70\cdot 10^{-7}$, respectively*.

#### 2.2.2. The Twirling-Whirling Instability

Consider an elastic rod of length *L* in a viscous fluid that at one end is forced to rotate about its long axis with frequency ω_0_ and the other end is free. Upon increasing the driving frequency the twirling regime of the rod switches to the whirling regime and the initially straight rod buckles. This elasto-hydrodynamic instability occurs at the critical frequency (Wolgemuth et al., 2000; Powers, 2010)

(15) $$\omega_{c}\approx 8.98\frac{A}{\zeta_{r}L^{2}},$$

where ζ_*r*_ is the rotational drag coefficient (torque per unit length). At low angular frequency ω_0_ < ω_*c*_, the steady-state twist distribution (i.e., the twist angle per unit length of the filament) along the straight filament of length *L* reads (Wolgemuth et al., 2000; Powers, 2010),

(16) $$\tau \left(s\right)=\frac{\zeta_{r}\omega_{0}}{C}\left(s-L\right),$$

where *s* is the arc-length parameter.

**Figure 2:**
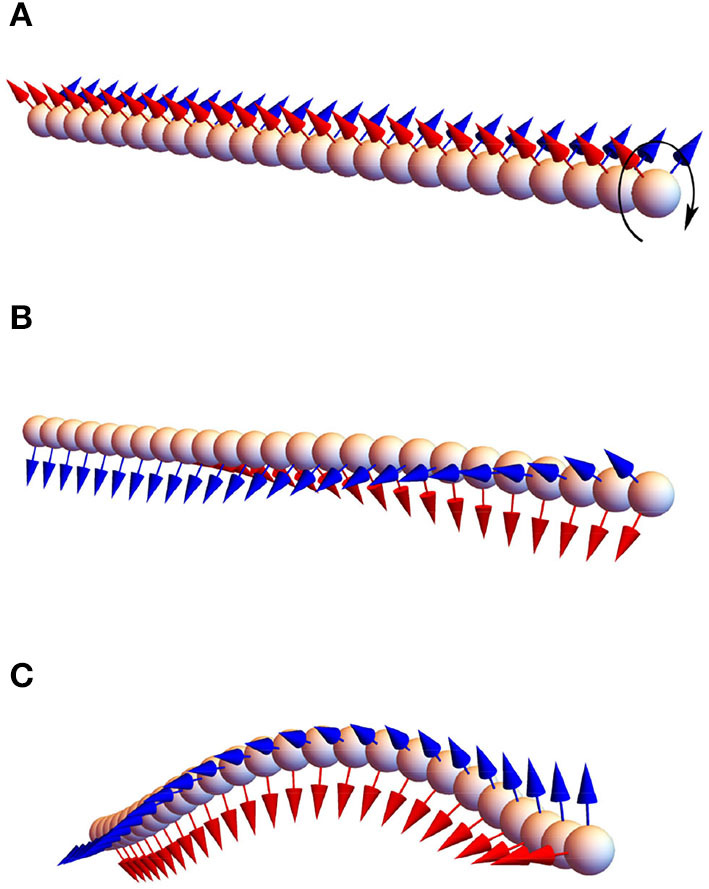
The twirling-whirling instability: an initially straight flexible 25-bead filament with *C*_*b*_ = 0.5 immersed in a viscous liquid and rotated at constant rate ω_0_ at one end, while the other end is free **(A)**; the filament buckles at the critical frequency ω_0_ = ω_*c*_ **(B)** acquiring the steady-state shape at higher frequency, ω_0_ > ω_*c*_ **(C)**. The color arrows stand for the binormal and normal vectors, *b*_*i*_ and *n*_*i*_.

We simulated the twirling-whirling instability numerically using bead-spring model as illustrated in Figure 2. Substituting *p* into Equation (16), the expression $\partial \tau /\partial s=\zeta_{r}\pi p/\eta a^{4}$ is obtained. In the calculation the value of $\zeta_{r}\approx 10\eta a^{2}$ was used (Morozov and Leshansky, 2014). Table 2A shows an excellent agreement of the numerically determined values of the angle (∂τ/∂*s*)*L*^2^ for a 25-bead filament at several driving frequencies ω_0_ < ω_*c*_ to the analytical prediction in Equation (16). Substituting the critical frequency ω_*c*_ into (16) and integrating over the filament length leads to the critical twist angle between its ends, Δφ_*c*_ = 4.49*C*_*b*_. The instability onset can be detected either by a sudden take-off of the bending energy or the maximum of the torsional energy. In the simulations *p* was gradually incremented until the torsional buckling (or whirling) of the filament occurs. Table 2B shows the critical value Δφ_*c*_ of the twist angle between filament ends at which the twirling-to-whirling transition occurs for a filament made of 25 beads, as calculated by Equation (15) and by using the bead-spring model upon varying *C*_*b*_ for a fixed value of *C*_*st*_ = 20. The agreement between the numerical results and the theoretical prediction is quite accurate, given the discrete nature of the bead-spring model. Figure 2B illustrates the near-critical shape of the filament at ω ≈ ω_*c*_, while Figure 2C depicts the steady-state shape of the whirling filament at ω_0_ > ω_*c*_ (see Supplementary Video 1).

**Table 2A:** The steady-state twist distribution along the axis of a 25-bead filament at low driving frequencies ω_0_ < ω_*c*_.

***p* (×10^5^)**	**(∂*τ*/∂*s*)*L*^2^ (rad)**	
	**Equation (16)**	**This work**
0.080	0.063	0.062
0.159	0.125	0.125
0.318	0.250	0.249

**Table 2B:** The critical twist angle between the filament ends, Δφ_*c*_, at the twirling-to-whirling transition.

***C*_*b*_**	**Critical twist angle, Δφ_*c*_(rad)**	
	**Theory**	**This work**
0.1	0.45	0.49
0.2	0.90	0.98
0.5	2.25	2.46
0.7	3.14	3.10
0.8	3.59	3.45
0.9	4.04	3.79
1.0	4.49	3.94
1.1	4.94	4.43

*The numerical results obtained for the 25-bead filament for several values of C_b_ = A/C are compared with the theoretical prediction, Δφ_c_ = 4.49C_b_*.

### 2.3. The Two-Segment Nanowire Propeller

Pak et al. (2011) reported an experimental design of a flexible nanomotor that displays high propulsion speed. This nanowire propeller, composed of a 1.8 μm-long rigid magnetic head (1.5 μm nickel segment and 0.3 μm gold segment) with a diameter of 200 nm and a 4 μm-long flexible silver tail with a diameter of ~100 nm, was actuated by the *conically* rotating magnetic field *H* = *H*_0_(*h*cosω*t*, −*h*sinω*t*, 1) where *h* = *H*_1_/*H*_0_ is the ratio of the rotating and constant components of the magnetic field whereas tan^−1^*h* is the cone angle.

Experiments were performed to determine the dependence of the nanomotor propulsion speed on the *sperm number* (Pak et al., 2011),

(17) $$\text{Sp}=L{\left(\frac{f_{⊥}\omega }{A}\right)}^{1/4},$$

for several values of the field cone angle upon varying *h*. Here *L* is the length of the nanowire's flexible part and *f*_⊥_ is the normal viscous drag coefficient (force density), given approximately for a slender filament by $f_{⊥}=\frac{4\pi \eta }{\log\left(2/\epsilon \right)+1/2}$, where ϵ = 2*a*/*L* ≪ 1 is the filament aspect ratio. In Pak et al. (2011) the value of the elastic constant *A* ≈ 3.6 × 10^−24^ N·m^2^ was best fitted to match the prediction of the approximate geometric theory assuming that the freely suspended nanowire can be approximated by an elastic filament with its one (clamped) end undergoing rotation with the cone angle equal to tan^−1^*h*.

Using the method described in this work we simulated the nanowire propulsion assuming an initially straight filament, composed of a 5-bead rigid head and a 11-bead flexible tail (i.e., a head-to-tail ratio being similar to the experiment in Pak et al., 2011), with the magnetic moment of the head set along its longitudinal axis. Notice that simulations of very slender filaments (with ϵ ≈ 0.025 as in Pak et al., 2011) would require large number of beads and are computationally expensive. As one may expect for viscous hydrodynamics, the larger dimension (i.e., the length *L*) of the slender nanowire is important, while its diameter (or the aspect ratio ϵ) has a minor effect on the dynamics (as can also be seen from the definition of Sp in Equation 17). Therefore, we expect that using of a chubby nanowire with ϵ ≈ 0.09 would yet closely approximate the dynamics of a slender filament in experiments of Pak et al. (2011).

**Figure 3:**
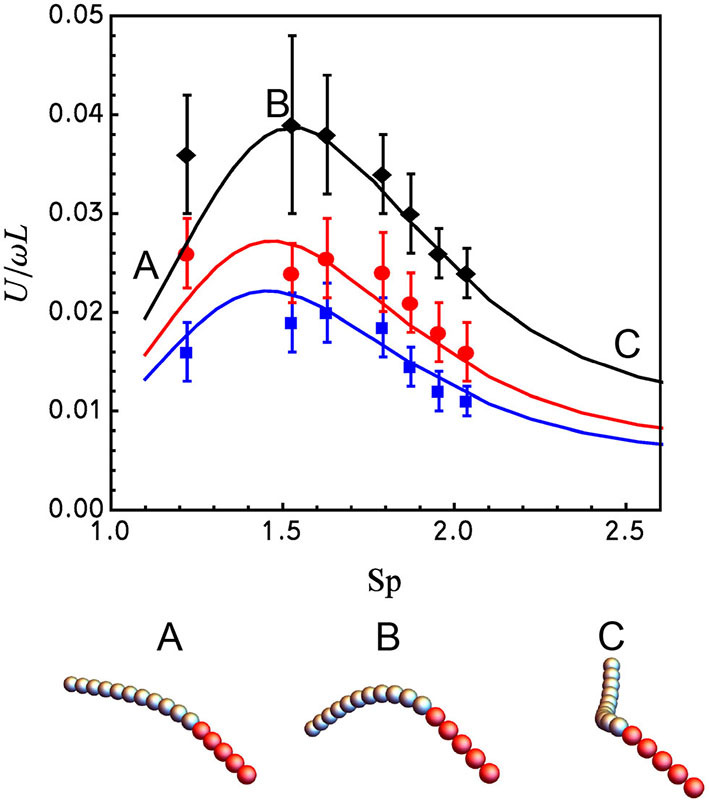
Dependence of the dimensionless swimming speed *U*/ω*L* of the 2-segment magnetic nanowire on the sperm number, Sp (upper panel). The experimental data (symbols), taken from Pak et al. (2011), and the numerical results for the bead-spring model (solid lines), are compared for three different actuating magnetic fields with *h*^−1^ = 1.43 (blue); *h*^−1^ = 1.18 (red); and *h*^−1^ = 0.70 (black). The lower panel shows the steady shapes of the simulated nanowire made of an 11-bead flexible tail (Ag, gray) and a 5-bead rigid magnetic (Ni, red) head magnetized longitudinally for *h*^−1^ = 0.70 and Sp = 1.1 (A), Sp = 1.5 (B), and Sp = 3.3 (C).

The dimensionless parameters were set to *C*_*m*_ = *C*_*b*_ = 1, *C*_*st*_ = 10 with *p* varying between 6·10^−7^ and 1.3·10^−4^. Figure 3 shows the dimensionless velocity, normalized by the length of the flexible tail, *L*, as a function of the sperm number Sp in (17) that can be rewritten in terms of dimensionless parameters as

(18) $$\text{Sp}=2\epsilon^{-1}{\left(\frac{\pi f_{⊥}p}{\eta C_{b}}\right)}^{1/4}.$$

**Figure 4:**
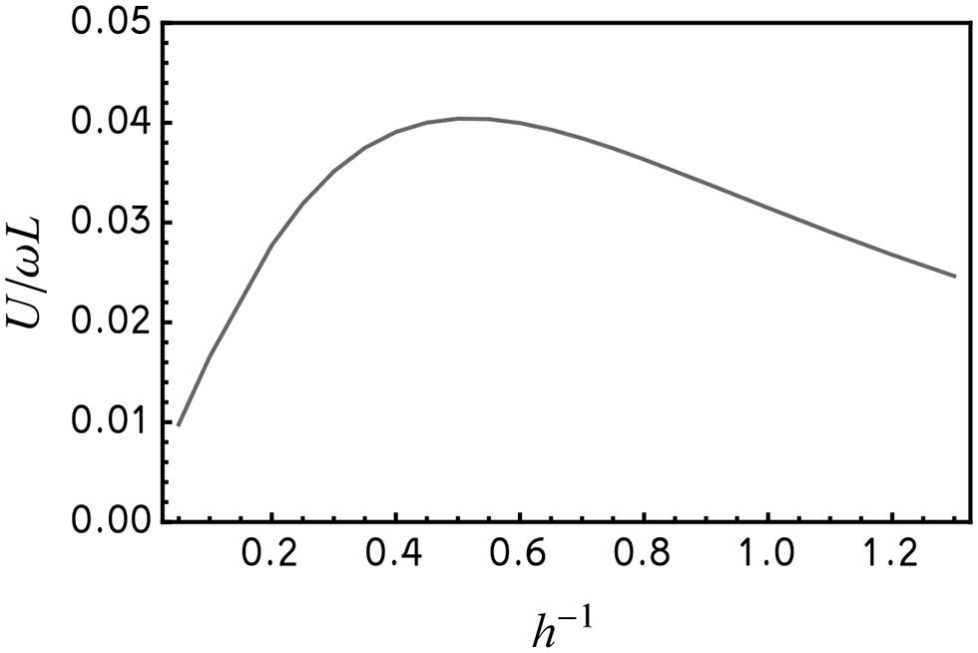
The dependence of dimensionless velocity *U*/ω*L* on *h*^−1^ at the (near) optimal value of Sp = 1.55. The data is based on the simulation of a nanowire propeller in Figure 3.

Best fitting of the numerical predictions to the experimental results requires ≈2.3 times larger value of the bending modulus, *A* ≈ 8.3 × 10^−24^ N·m^2^, when compared to the estimate of Pak et al. (2011). It can be readily seen that the agreement between the numerical predictions and the experimental findings in Figure 3 is excellent without any extra adjustable parameters^1^. In particular, the maximum speed is attained at Sp ≈ 1.55 (see Supplementary Videos 2–4). The numerical method allows for finding the optimal value of *h*^−1^ maximizing the propeller's velocity. Computing the velocity of the propeller at the optimal sperm number (Sp ≈ 1.55) reveals that the optimal value of *h*^−1^ is ≈ 0.5, meaning the magnitude of the constant magnetic field is half the magnitude of the rotating field (see Figure 4).

### 2.4. The Three-Segment Nanowire Propeller

Gao et al. (2010) demonstrated the propulsion of a nanowire motor with magnetic nickel head and golden tail (both rigid segments) connected by a flexible link made of porous silver. In the experiments they found that upon applying an external rotating magnetic field, two propulsion gaits are observed: slow backward motion (i.e., toward the passive tail), and fast forward motion (toward the magnetic head). We simulated this nanowire as a filament made of three segments each composed of 5, 10, and 5 beads, corresponding to the magnetic head, flexible link and passive tail, respectively. All simulations of the initially linear filaments resulted in the forward propulsion gait regardless of the magnetic moment orientation. However, it was found that some imperfection in the initial shape of the filament (e.g., slight intrinsic curvature of the Ni-head) the bidirectional propulsion gait can be realized in a qualitative agreement with the experiments by Gao et al. (2010), where the microfabricated nanowires at rest were not straight.

**Figure 5:**
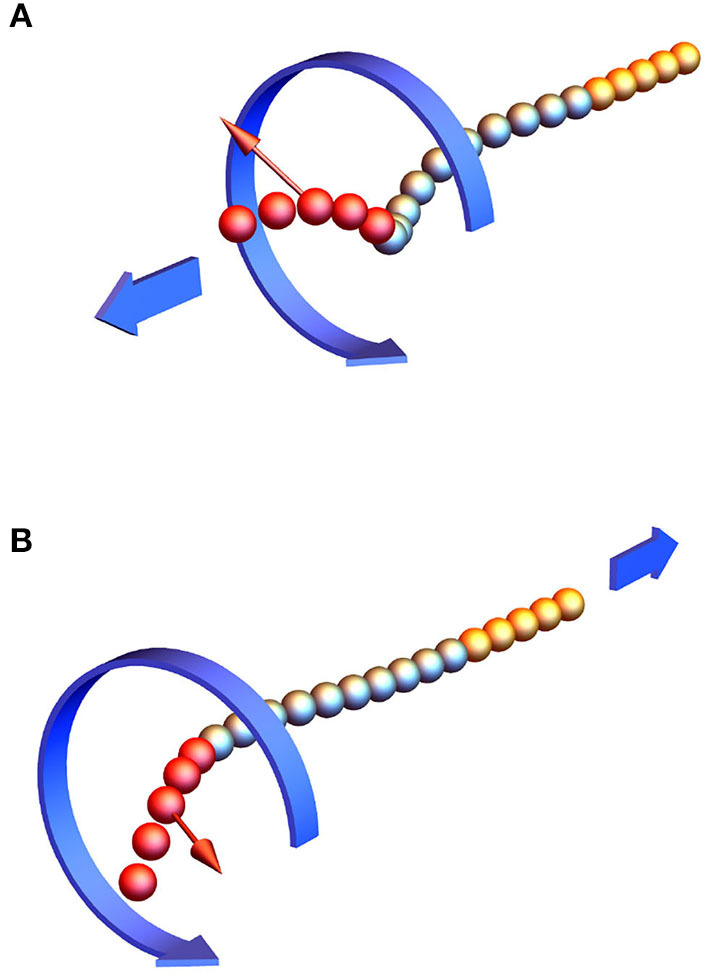
The simulated three-segment nanowire, composed of Au rigid tail (yellow), a flexible middle Ag link (gray) and a slightly curved rigid magnetic Ni head (red). Two realizations of the nanowire were simulated, both have the same magnetization component in the swimmer's plane, whereas their off-plane components of magnetization have the same magnitude, but opposite sign. **(A)** The steady-state 3D shape of the nanowire when propelling head-forward (fast); **(B)** The steady-state shape of the nanowire when propelling tail-forward (slow).

Morozov et al. (2017) predicted and it was later demonstrated experimentally (Sachs et al., 2018) that rigid planar objects (such as arc) can change their propulsion direction when the sign of the off-plane magnetization component is reversed. Similarly, it was observed in the simulation of the three-segment nanowire that reversal the off-plane component of the head's magnetic moment, results in propulsion reversal, i.e., switches between fast head-forward and slow tail-forward motion (see videos #5 and #6 in the Supporting Materials). In both cases the magnetic head follows the rotation of the actuating field. Figures 5A,B depict the nanowire steady-state shapes for both propulsion gaits. Setting the dimensionless parameters to *C*_*m*_ = *C*_*b*_ = 1, *C*_*st*_ = 10, and *p* = 10^−4^, the forward and backward dimensionless speeds *U*/*Lω* (here *L* is the length of the flexible part) were determined numerically to be 0.0011 and 0.0084, respectively, implying that the head-forward motion is about 8 times faster. These findings are in a qualitative agreement with the experimental observations of Gao et al. (2010).

## 3. Conclusions

We developed a numerical scheme based on a discrete bead-spring model for simulating soft nanowire-based propellers. The algorithm was favorably tested by simulating a well-known twirling-whirling instability of an elastic filament rotated in a viscous liquid. The comparison of the numerical prediction for the propulsion speed vs. the reported experimental results for the Ni-Ag nanowire actuated by a conically rotating magnetic field in Pak et al. (2011) as a function of actuation frequency (Sp) upon varying the ratio of the in-plane rotating and constant field components (*h*^−1^), showed an excellent agreement (see Figure 3). At moderate actuation frequencies the Ni-Ag nanowire adopts the arc shape, while it develops 3D geometric chirality gradually as Sp increases above ≈ 2.5 The simulation results predict an optimal cone angle of the actuating field, ≈ 63°, that maximizes the value of the non-dimensional propulsion velocity *U*/ω*L* at Sp ≈ 1.5. Surprisingly, at the optimum the nanowire has a shape of a planar arc (see Figure 3B), indicating that efficient propulsion of magnetically driven soft nanowires does not require 3D geometric chirality as was previously suggested. This finding is in accord with Mirzae et al. (2018) where an efficient steering of rigid magnetic arc-shaped nanomotors was demonstrated.

Using our model we could substantiate important and previously unexplained details, such as bidirectional (fast head-forward and slow tail-forward) propulsion of the Ni-Ag-Au nanowire of Gao et al. (2010) powered by in-plane rotating magnetic field due to potential shape imperfection which is probably inevitable in nanowire nanofabrication. The proposed numerical model can be used as an efficient tool for design and optimization of previously proposed and also novel soft nanomachines. It can be also extended to modeling of polarizable (superparamagnetic) soft nanowires, biological and biohybrid microswimmers and other externally or self-propelled machines.

## Data Availability Statement

The original contributions generated for the study are included in the article/Supplementary Material, further inquiries can be directed to the corresponding author/s.

## Author Contributions

YM implemented the bead-spring algorithm and performed all the computations. BR and KM assisted in developing the key ideas, research design, and implementation. AL directed the research and wrote the paper. All authors contributed to the article and approved the submitted version.

## Conflict of Interest

The authors declare that the research was conducted in the absence of any commercial or financial relationships that could be construed as a potential conflict of interest.

## Footnotes

^1^In Pak et al. (2011) the results shown by black diamonds in Figure 3 correspond to *h*^−1^ = 0.95, while our numerical simulations suggest that the correct value is actually close to *h*^−1^ = 0.7.

## Supplementary Material

Supplementary Video 1 Twirling-whirling instability of an elastic filament for *C*_*b*_ = 0.5.

Supplementary Video 2 The two-segment (Ni-Ag) nanowire propeller at Sp = 1.1.

Supplementary Video 3 The two-segment (Ni-Ag) nanowire propeller at Sp = 1.5.

Supplementary Video 4 The two-segment (Ni-Ag) nanowire propeller at Sp = 3.3.

Supplementary Video 5 Head-forward (fast) motion of the three-segment (Ni-Ag-Au) nanowire propeller.

Supplementary Video 6 Tail-forward (slow) motion of the three-segment (Ni-Ag-Au) nanowire propeller.
